# Band Engineering via Charge Modulation Drives High Thermoelectric Performance in Conductive MOFs

**DOI:** 10.1021/acs.jpclett.6c01808

**Published:** 2026-07-17

**Authors:** Hardik L. Kagdada, R. Dettori, L. Colombo, C. Melis

**Affiliations:** Department of Physics, University of Cagliari, Monserrato, CA 09042, Italy

## Abstract

Metal–organic frameworks (MOFs) are promising thermoelectric materials due to their low lattice thermal conductivity and tunable electronic properties. However, many conductive MOFs exhibit metallic behavior, leading to low Seebeck coefficients and high electronic thermal conductivity, which limit thermoelectric efficiency. We propose a band-engineering approach based on controlled charge modulation to tune the electronic structure by modifying the oxidation state of the metal centers. Using copper benzenehexathiol (CuBHT) as a model MOF, density functional theory, electron-phonon coupling, and Boltzmann transport calculations show that adjusting Cu^+^/Cu^2+^ states triggers a metal-to-semiconductor transition with a band gap, boosting the Seebeck coefficient and reducing electronic thermal conductivity. This increases *zT* by nearly 2 orders of magnitude. Further, optimization via 5% compressive strain increases the Seebeck coefficient to about 250 μV K^–1^ at 300 K (10 times higher than pristine) and achieves a maximum *zT* of 0.79 at 550 K, approaching the performance of established thermoelectric materials and surpassing most reported MOF-based systems.

## Graphical abstract

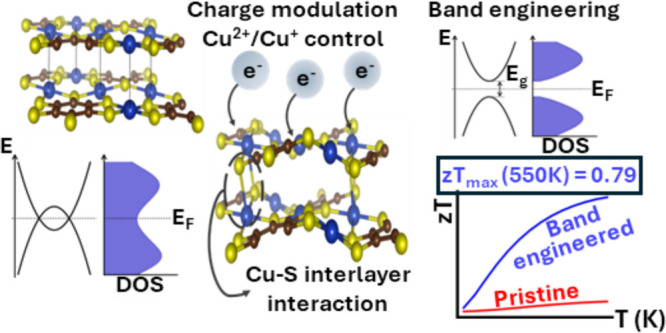

Thermoelectric materials enable the direct conversion of heat into electricity under a temperature gradient and are attracting increasing attention for waste-heat recovery and solid-state energy conversion technologies.^1,2^ Their efficiency is governed by the dimensionless figure of merit

1 $$zT=\frac{S^{2}\sigma T}{\kappa }$$

where *S* is the Seebeck coefficient, σ is the electrical conductivity, and κ = κ_l_ + κ_e_ is the total thermal conductivity, including both lattice (κ_l_) and electronic contributions (κ_e_). Maximizing *zT* remains intrinsically difficult because these transport coefficients are strongly coupled: increasing carrier concentration generally enhances σ but often suppresses *S*, while high electrical conductivity is usually associated with a larger κ_e_ through the Wiedemann–Franz relation.^3,4^

Metal–organic frameworks (MOFs) are attractive in this context because they naturally combine structural complexity, low mass density, soft bonding, and internal porosity, all of which are highly favorable for suppressing phonon transport.^5–7^ Experimentally, many MOFs exhibit room-temperature thermal conductivities well below those of conventional inorganic thermoelectrics, often in the range ∼0.1 –1 W m^–1^ K^–1^, with representative values such as 0.11 W m^–1^ K^–1^ for UiO-66 powders, 0.19 W m^–1^ K^–1^ for UiO-67 powders, and 0.26–0.73 W m^–1^ K^–1^ for HKUST-1 depending on morphology and measurement conditions.^7^ Conductive MOFs as well offer unusual chemical flexibility: metal node, linker frontier orbitals, stacking arrangement and degree of π–*d* conjugation can all be adjusted to tune band dispersion, carrier density, and anisotropy in charge transport.^8–11^ In summary, the combination of ultralow lattice thermal conductivity and a chemically tunable electronic structure makes conductive MOFs, particularly two-dimensional systems, highly appealing for approaching the phonon-glass electron-crystal paradigm.^12–14^ Several 2D conjugated MOFs and coordination polymers display electrical conductivities ranging from semiconducting values up to metallic-like levels approaching 10^3^ S cm^–1^, while maintaining lattice thermal conductivities below 1 W m^–1^ K^–1^.^8,9,15,16^ These characteristics suggest that MOFs could, in principle, decouple heat and charge transport more effectively than many dense inorganic solids.

In practice, however, the thermoelectric performance of conductive MOFs has so far remained (at least) modest. Reported room-temperature *zT* values for intrinsic conductive MOFs are typically in the 10^–3^–10^–2^ range. For example, Ni_3_(HITP)_2_ combines an ultralow thermal conductivity of about 0.2 W m^–1^ K^–1^ with a record MOF *zT* of order 10^–3^.^13^ Similarly, the perthiolated-coronene-based framework [Ni_3_(C_24_S_12_)]_*n*_ shows *S* = 47 μV K^–1^, κ = 0.2 W m^–1^ K^–1^, and *zT* = 0.003 at 300 K.^17^ More recently, improved film formation and host–guest control in nickel–nitrogen-based quasi-2D conjugated coordination polymers increased *zT* to 8.1 × 10^–3^, highlighting that progress is possible but still far from the performance of established inorganic thermoelectrics.^18^ Theoretical studies have predicted that selected 2D MOFs could approach or exceed *zT* ∼ 0.1 under optimized electronic conditions, highlighting their strong potential for thermoelectric applications, although experimental validation is still developing.^9,19^ The main reason for such a poor thermoelectric performance is that the most conductive MOFs frequently exhibit metallic or quasi-metallic transport. This is beneficial for σ, but detrimental to the Seebeck coefficient because a high carrier concentration and a weak energy dependence of the conductivity around the Fermi level suppress *S*.^3,9,20^ In addition, once the system exhibits strong metallic character, the electronic contribution to the thermal conductivity can no longer be neglected and may reduce the benefit of an ultralow κ_l_.^2,15^ Additional factors can further depress the power factor (PF), including multiband compensation, Fermi-level pinning in nearly symmetric bands, disorder-induced carrier localization, and microstructural effects such as grain boundaries and imperfect stacking, which are especially relevant in polycrystalline 2D films.^8,9,14^ As a result, many conductive MOFs only partially meet the thermoelectric requirement—low thermal conductivity—without simultaneously achieving the band asymmetry and carrier optimization needed for large *S* ^2^σ. This limitation is particularly severe in copper benzenehexathiol (CuBHT or Cu_3_BHT), a prototypical 2D conductive MOF that has attracted broad interest.^14,15,21^ Recent studies report lattice thermal conductivity in the ultralow range of ∼0.2 –1 W m^–1^ K^–1^ together with electrical conductivity up to 2000 S cm^–1^, emphasizing the exceptional decoupling between charge and heat transport in this system.^15,21^ However, pristine CuBHT generally retains a strongly metallic or quasi-metallic electronic character, which leads to low Seebeck coefficients and a sizable κ_e_, ultimately limiting *zT* despite the favorable phonon transport background.^14,15^

A promising route to overcome this bottleneck is to act directly on the electronic state through redox or charge control rather than relying only on structural perturbations. This idea is particularly well motivated in benzenehexathiol-based copper frameworks, where recent experiments have shown that CuBHT films synthesized by liquid–liquid interfacial reaction exhibit a fractional Cu oxidation state resulting from an intramolecular pseudoredox mechanism between Cu^+^/Cu^2+^ centers and BHT ligands.^16,22,23^ In those experiments, CuBHT is grown at the interface between CuSO_4_/H_2_O and BHT/toluene, and spectroscopic analyses indicate that the Cu valence state is intimately linked to the electronic structure of the film.^16^ Interestingly, the same study showed that reducing the Cu^2+^/Cu^+^ ratio induces band gap opening in CuBHT thin films, while thickness-dependent growth determines whether the system remains semiconducting or evolves toward a metallic phase with interlayer Cu–S bonds.^16^ These results indicate that oxidation-state control provides a chemically accessible route to tune transport in CuBHT-based systems.

In this proof-of-concept work, we exploit this approach by adopting a band-engineering strategy based on controlled charge modulation to directly tune the Cu^+^/Cu^2+^ balance and to modify the electronic structure of CuBHT. Using first-principles calculations combined with electron–phonon coupling and Boltzmann transport theory, we show that charge modulation induces a metal-to-semiconductor transition with band gap opening. This leads to a strong increase in the Seebeck coefficient and a strong reduction in the electronic thermal conductivity. We further provide evidence that moderate compressive strain represents an additional degree of control over band dispersion and carrier transport, leading to a further improved thermoelectric performance. The resulting *zT* values, approaching ∼0.8 at intermediate temperature, are orders-of-magnitude larger than those typically reported for MOF-based thermoelectrics and identify oxidation-state engineering as a viable design principle for high-performance conductive MOFs.

The article is organized as follows. We first discuss the properties of pristine CuBHT, assess the effect of structural perturbations and controlled charge modulation, and finally examine strain engineering as an additional strategy to optimize the thermoelectric performance. We then discuss the computational framework used to evaluate the electronic structure, carrier lifetimes, and thermoelectric transport coefficients. Further computational details, additional band structures, transport data, and Bader charge analysis are reported in the Supporting Information (SI).

The CuBHT structure considered here is characterized by an AA stacking sequence, corresponding to a pseudorhombohedral arrangement in which the Cu–S kagome layers are nearly hexagonal but formally belong to a lower triclinic symmetry (Figure 1a). The layers are superimposed along the out-of-plane direction, preserving the in-plane Cu–S network. The computed lattice parameters (*a* = 8.659 Å, *b* = 8.682 Å, *c* = 3.401 Å, α = 72.11°, β = 90.28°, γ = 119.95°) are in very good agreement with experimental reports for well-ordered films and single crystals (*a* = *b* = 8.675 Å, *c* = 3.489 Å),^16^ confirming the reliability of the optimized geometry. Minor deviations in angular parameters arise from the pseudorhombohedral distortion and the use of a fully relaxed triclinic cell. The small contraction of lattice constants relative to some previous theoretical estimates can be attributed to the use of the PBEsol functional with van der Waals corrections and a higher plane-wave cutoff, which provide an improved description of interlayer interactions.^24^ The calculated out-of-plane lattice parameter is consistent with TEM measurements for multilayer stacks,^25^ supporting the presence of weak interlayer coupling in the absence of axial Cu–S contacts. Experimentally, multilayer CuBHT films have also been reported to remain thermally stable up to approximately 750 K.^26^ The electronic band structure and density of states (DOS) of CuBHT indicate metallic behavior, as the valence and conduction bands cross the Fermi level (Figure 1b). We also computed the electronic band structure using the HSE06 hybrid functional. However, the metallic nature persists (see Figure S1). The projected density of states (PDOS) near the Fermi level shows that Cu and S atoms contribute most to the total DOS. The relaxation time (τ) is computed by thoroughly accounting for electron–phonon scattering processes.

**Figure 1:**
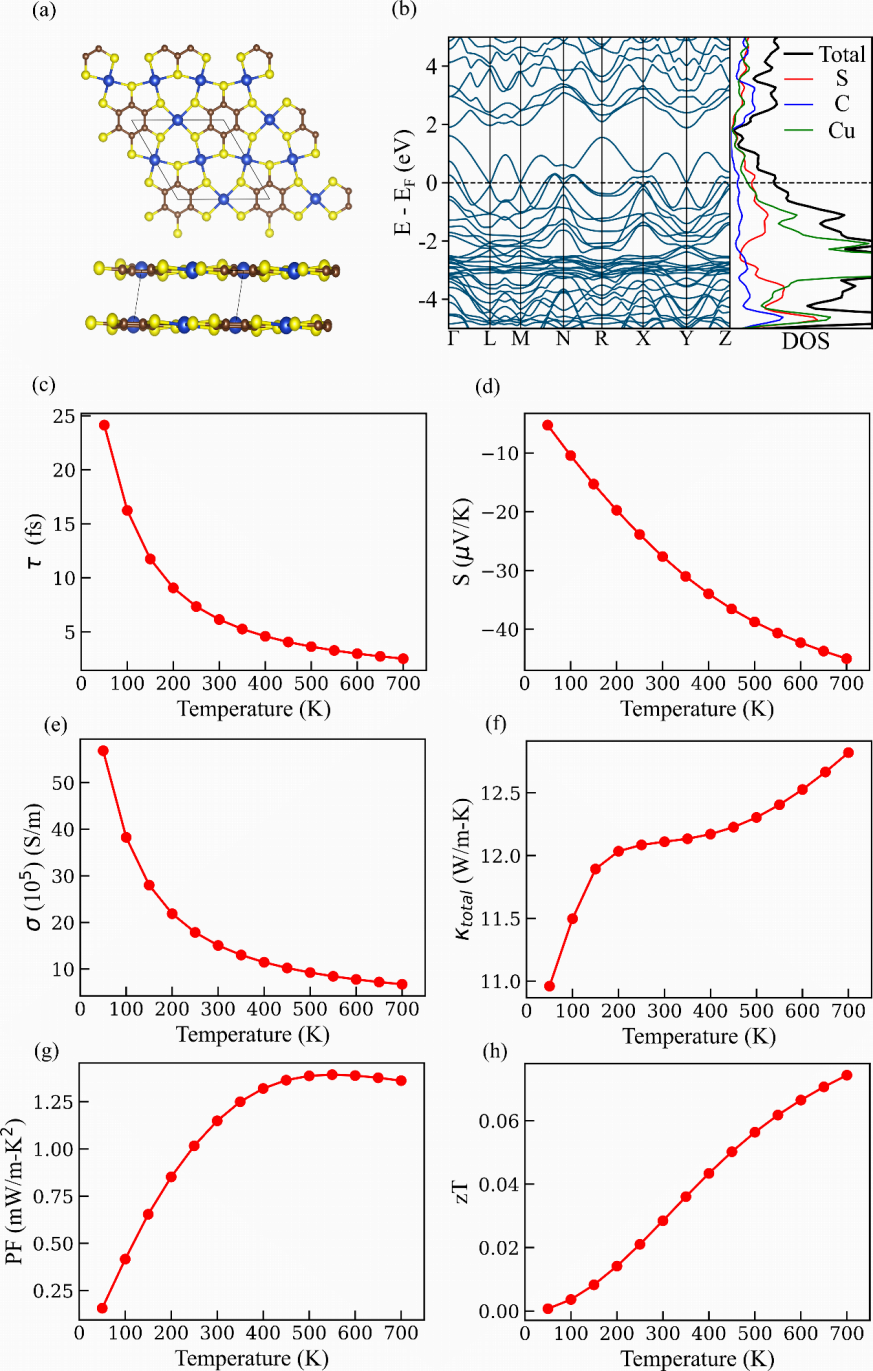
(a) Ball-and-stick model of the AA-stacked CuBHT crystal structure, shown from top and side views. Carbon, sulfur, and copper atoms are represented in brown, yellow, and blue, respectively. (b) Electronic band structure and elemental DOS for pristine CuBHT, with the Fermi level set to 0 eV and indicated by a horizontal dashed line. (c) τ computed from electron–phonon interactions. In-plane transport coefficients, (d) *S*, (e) σ, (f) total thermal conductivity (κ_total_), (g) power factor (PF), and (h) *zT* for pristine CuBHT. The figure of merit shows a strong correlation with *S*. The lattice thermal conductivity used in the calculation of κ_total_ was obtained from previously reported values by this group.^21^

For pristine CuBHT, the temperature-dependent average τ is plotted in Figure 1c. Although the value of τ at 300 K (6.08 × 10^–15^ s) is shorter than the $∼41\times 10^{-15}$ s scattering time reported in a previous experimental work,^16^ the difference is expected because the two values refer to distinct transport regimes and methodologies. Our relaxation time is obtained from first-principles electron–phonon scattering for equilibrium carriers at the Fermi level in metallic CuBHT, where the high DOS enhances scattering and reduces carrier lifetimes. In contrast, the experimental value represents an effective momentum-averaged scattering time of photoexcited band-edge carriers in semiconducting thin films, extracted from a Drude-model fit, and therefore is not directly comparable to the microscopic lifetime calculated here.

The metallic nature of pristine CuBHT results in a high in-plane conductivity of 1.5 × 10^6^ S/m at 300 K (Figure 1e), while the in-plane *S* remains small (−27.62 μV K^–1^), as expected for a high carrier-density system (Figure 1d). This value is in agreement with the experimental measurements reported by Tsuchikawa et al. for polycrystalline CuBHT thin films, where *S* ranges from −10 to −21 μV K^–1^ at 300 K.^14^ In that work, the small magnitude of *S* was attributed to the intrinsic metallic character of crystalline CuBHT domains, despite the presence of polycrystallinity and percolative transport effects. By contrast, smaller *S* values ( ∼2 –8 μV K^–1^) reported in more recent studies of highly conductive CuBHT films^15^ have been associated with ambipolar transport and partial electron–hole compensation, which further suppresses the thermopower. The consistency between our calculated value and the experimentally observed metallic range supports the reliability of the electronic structure description and confirms the metallic character of the AA-stacked phase. The magnitude of the Seebeck coefficient |*S*| increases monotonically with temperature (Figure 1d), while the electrical conductivity σ decreases, rapidly up to 600 K and more gradually at higher temperatures (Figure 1e), following the trend of τ. The total thermal conductivity, dominated by the electronic contribution, increases with temperature. The electronic thermal conductivity κ_*e*_ = 12.11 W m^–1^ K^–1^ is about 1 order of magnitude larger than the lattice contribution ( ∼1.31 W m^–1^ K^–1^).^21^ This behavior is consistent with the Wiedemann–Franz law, with a Lorentz number *L* = 2.69 × 10^–8^ V^2^ K^–1^, close to the ideal value *L*_0_ = 2.44 × 10^–8^ V^2^ K^–1^, indicating that κ_*e*_ scales approximately with *σT* as expected for a metallic system. The computed κ_total_ at 300 K (Figure 1f) is significantly higher than the experimental value of ∼1.1 W m^–1^ K^–1^.^15^ This difference mainly arises from the much larger electrical conductivity predicted for the ideal AA-stacked structure (1.5 × 10^6^ S m^–1^) compared to experimental values ( $∼2\times 10^{5}$ S m^–1^)^15^ and the defect-free situation considered in present calculations. In experiments, CuBHT thin films exhibit structural disorder and paracrystallinity (>10%) , which reduce σ and, consequently, the electronic contribution to thermal conductivity.

Considering all transport parameters, the calculated *zT* at 300 K for pristine CuBHT is 0.028 (Figure 1f), about 1 order of magnitude higher than the experimental value reported by Un et al. (∼0.0015)^15^ and within the same range as the value of ∼0.013 reported by Tsuchikawa et al.^14^ The differences among the reported experimental *zT* values likely reflect the strong sensitivity of CuBHT transport properties to microstructure, grain boundaries, stoichiometry, and structural disorder, as highlighted by recent experimental studies.

We investigate whether structural or charge density modifications can modify the metallic character of pristine CuBHT. Previous studies have suggested that structural disorder (e.g., strain and vacancy defects) and charge modulation are indeed effective strategies to enhance the power factor (PF).^1,3,4,27^ To assess whether structural modifications alone can modify the metallic character of pristine CuBHT, we examined the effects of 5% uniaxial strain (along the *a* and *b* axes), biaxial strain (in the *ab* plane), and vacancy defects on its electronic band structure. Vacancy defects were introduced by removing one or two Cu or S atoms from the unit cell (denoted as 1-Cu­(S) and 2-Cu­(S), respectively). Removing one Cu atom (1-Cu defect) reduces the Cu/BHT molar ratio (MR) from the ideal value of 3 in Cu_3_BHT to 2, corresponding to experimentally investigated compositions.^15^ However, in the present model, this change is introduced through a vacancy, which locally perturbs the lattice and can induce carrier scattering. In all cases, the band structure remains metallic under both compressive and tensile strain, as well as in the presence of vacancy defects (see Figure S2), indicating that structural disorder alone is insufficient to induce a metal-to-semiconductor transition. Accordingly, the temperature-dependent carrier relaxation time obtained for pristine CuBHT was used to estimate the transport properties of the strained and defective metallic phases.

The in-plane *S* is reduced in both strained (Figure S3) and defective structures (Figure S4), while the in-plane σ remains comparable under strain but decreases significantly in the presence of defects. This reduction is particularly pronounced for the 1-Cu defect. Although MR = 2 has been associated with more ordered films experimentally,^15^ in our case the decrease in σ may arise from defect-induced carrier scattering due to the vacancy.^28–32^ Overall, defects introduce carrier trapping effects that reduce σ and the PF, while strain alone has a limited impact on transport properties. Nevertheless, the calculated *S* and corresponding *zT* remain lower than in pristine CuBHT for both strain and defect engineering, limiting its thermoelectric performance. This behavior originates from the intrinsic metallic character of CuBHT, which persists under these perturbations. Therefore, opening a band gap is essential to enhance *S* and, consequently, *zT*.

In this context, recent work by Fu et al.^16^ suggests that controlling the Cu oxidation state provides an effective strategy to tune the electronic properties of CuBHT, enabling a transition from metallic to semiconducting behavior. In the same work, the authors investigated hot-carrier mobilities and charge transport mechanisms using a combined experimental and DFT-based computational approach. Their CuBHT thin films were synthesized via liquid–liquid interfacial growth, where early stage interfacial confinement promotes highly ordered AA stacking. X-ray absorption and photoemission spectroscopy revealed a fractional Cu oxidation state associated with a pseudoredox process involving the Cu^2+^/Cu^+^ redox pair. DFT calculations performed in the same study, employing a charge-modulation approach to mimic the Cu redox process, showed that reducing the Cu^2+^/Cu^+^ ratio from 1 to 0 induces a band gap opening ( ∼0.4 eV), consistent with optical absorption and conductivity measurements indicating semiconducting behavior (σ ≈ 48 S cm^–1^). These results suggest that controlling the Cu oxidation state, together with stacking effects, could stabilize a semiconducting phase in CuBHT.

Motivated by these findings, we systematically examined the impact of controlled charge modulation on the electronic and thermoelectric properties of CuBHT, following the DFT approach of ref.^16^ The addition of electrons is intended to mimic the partial reduction of Cu^2+^ to Cu^+^ reported experimentally, which modifies the Cu oxidation state and associated electronic structure. Adding three electrons per unit cell corresponds to about one extra electron per Cu atom, effectively mimicking the partial reduction of the Cu centers. We note that this computational approach represents an idealized electronic state, enabling the exploration of different electronic phases that may emerge under strong charge modulation (e.g., through redox control or electrochemical gating). Accordingly, the present computational model is not intended to predict a new thermodynamically stable charged crystal. Rather, it provides a first-principles proof-of-concept framework to investigate how experimentally motivated charge modulation affects the electronic structure and thermoelectric transport properties of CuBHT.

Introducing a charge of −3e (hereafter CuBHT-3e) induces a metal-to-semiconductor transition, opening a band gap of 0.05 eV (Figure 2(a)). In fact, pristine CuBHT is characterized by a gap in the conduction band below 2 eV (Figure 1b), and the addition of three electrons shifts the Fermi level further toward the conduction band and into this gapped region (Figure S5­(a)). We also performed electronic band structure calculations using the screened hybrid functional HSE06 ^33–35^ (Figure S5­(b)), which incorporates 25% Hartree–Fock exchange. Within the DFT+HSE06 framework, the band gap increases to 0.9 eV, consistent with trends reported in the literature.^16^ Our HSE06 band gap is higher than that reported by Fu et al.,^16^ likely due to differences in the van der Waals functionals employed. Bader charge analysis (Table S1) for both pristine and charged systems shows that the addition of electrons reduces the positive charge on both the Cu atoms and the BHT molecule. At a charge of −3e, a partial Cu^2+^ to Cu^+^ transition may occur, accounting for the observed band gap opening.^16^ However, the change in Cu charge suggests a partial and delocalized redox behavior rather than a complete Cu^2+^ to Cu^+^ transition, as the added electrons are distributed across all atoms (see Table S1). The three Cu atoms exhibit partial charges of −0.342e, −0.248e, and −0.191e, becoming less positively charged in the CuBHT-3e structure and indicating a tendency toward Cu^+^. The localization of a significant fraction of the excess charge on the BHT ligand, rather than on the Cu atoms, highlights the delocalized nature of the charge redistribution. Furthermore, orbital-projected electronic structure analysis shows that the valence-band maximum is primarily composed of bonding (Cu $d_{x^{2}-y^{2}}$ , *d*_*xy*_, and *d*_*yz*_) states hybridized with S (*p*_*x*_) and (*p*_*y*_) orbitals, while the conduction band is dominated by antibonding Cu (*p*_*z*_) and C (*p*_*z*_) states. As a result, the injected electrons occupy states with significant antibonding character, weakening the in-plane Cu–S bonding network that stabilizes the planar framework. On the other hand, the increased contribution of Cu (*d*_*yz*_) and (*p*_*z*_) orbitals promotes out-of-plane Cu–S interactions and drives the Cu centers toward a sulfur-coordinated octahedral-like environment. This process lowers the system’s total energy through an out-of-plane buckling distortion, characterized by a displacement of the sulfur atoms (approximately 0.6 Å) and the formation of interlayer Cu–S bonds (2.66 Å), while the in-plane Cu–S bonds remain largely preserved (Figure 2(b)). This behavior is consistent with the experimental observations of Fu et al.,^16^ who demonstrated that changes in the Cu^2+^/Cu^+^ ratio modify both the electronic structure and stacking configuration of CuBHT, including the emergence of interlayer Cu–S interactions.

**Figure 2:**
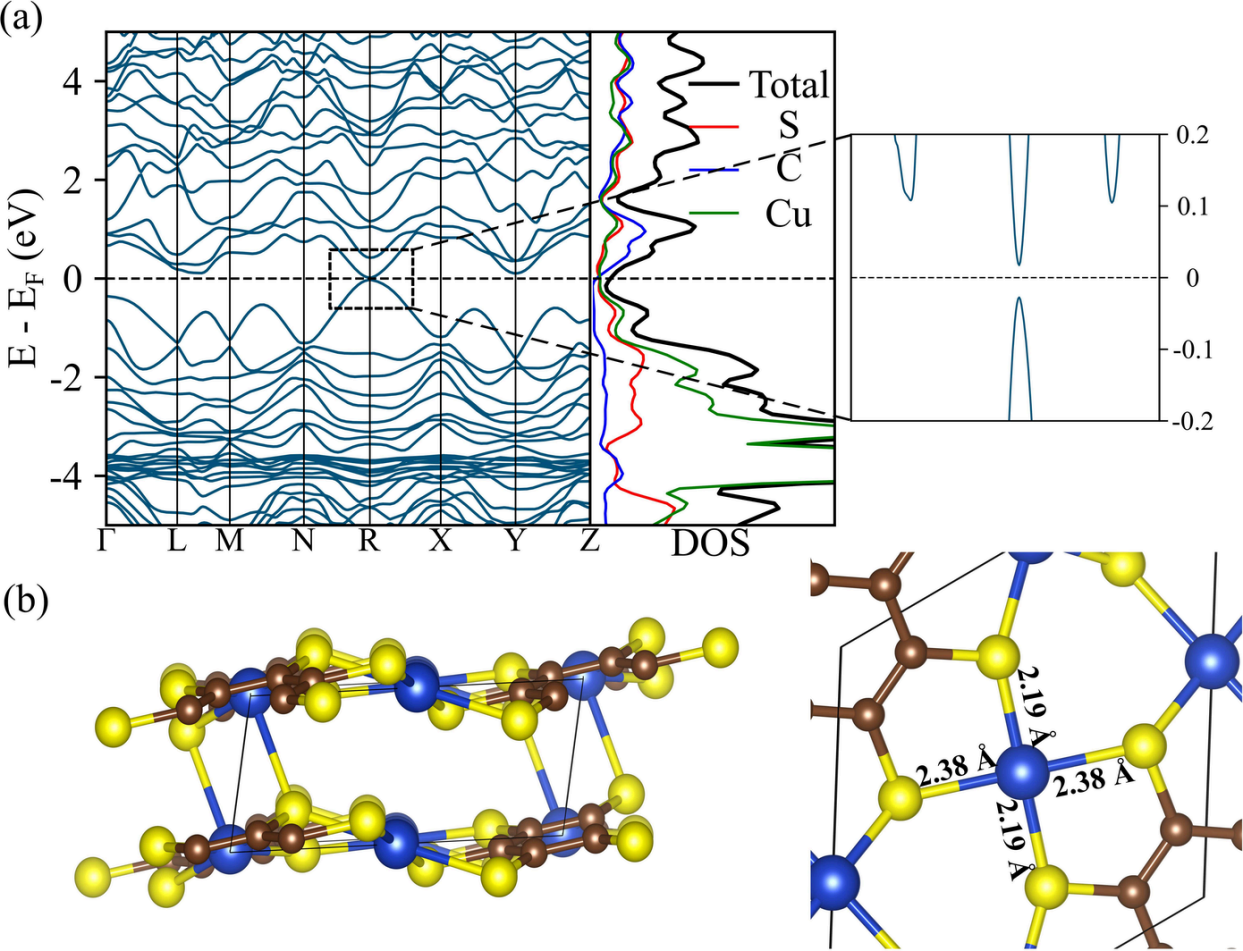
(a) Calculated electronic band structure and DOS for CuBHT with an additional −3e charge (CuBHT-3e). The inset on the right illustrates the band gap opening at the high symmetry point R in the first Brillouin zone. (b) Side view of the CuBHT-3e structure highlighting interplanar bonds between Cu and S atoms (left), and in-plane view emphasizing the Cu–S in-plane bond length.

We evaluated transport coefficients for a p-type carrier concentration of 10^20^ cm^–3^, corresponding to a representative degenerate doping regime commonly considered in thermoelectric materials. This choice allows us to explore the regime where the power factor is typically maximized. The S gradually increases with temperature, reaching a maximum value of 155 μV/K at 600 K (Figure 3a) (see Figure S6 for a representation of carrier concentration dependent thermoelectric parameters). At 300 K, the *S* of CuBHT-3e reaches 100 μV/K enhanced by a factor of 3 compared to pristine CuBHT. In the pristine case, the computed transport distribution function (TDF) for the metallic case remains finite and shows only a weak dependence on energy near the Fermi level due to its metallic nature. On the other hand, the TDF in CuBHT-3e vanishes within the bandgap region, showing a sharp onset and noticeable energy dependence near the Fermi level (Figure 3b). This strong energy dependence of the TDF arises from the sharp increase in the DOS near the Fermi level (Figure 3c).

**Figure 3:**
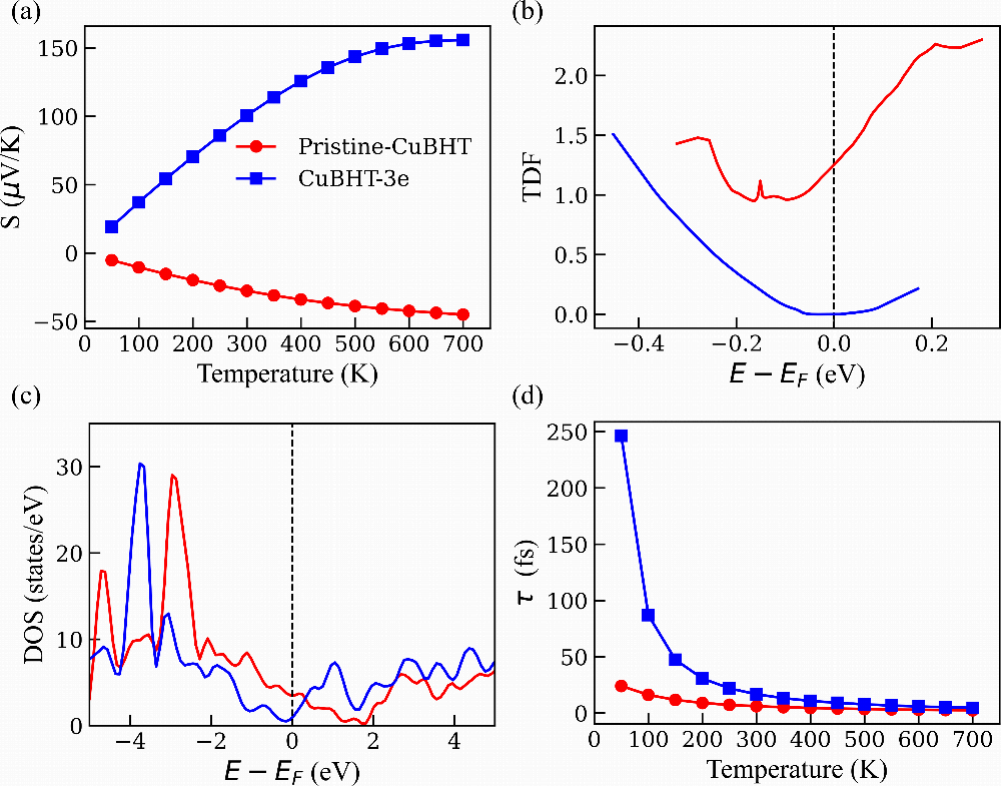
In-plane transport parameters, (a) *S*, (b) transport distribution function (TDF; see computational methods in the SI for its definition), (c) electronic DOS, (d) τ for CuBHT-3e and pristine CuBHT for 10^20^ cm^–3^ p-type carriers. For CuBHT-3e, the *S* increases by a factor of 3 at 300 K, reflecting its semiconducting behavior. Moreover, pristine CuBHT exhibits a weak energy dependence of the TDF due to its metallic character. Fermi energy is shifted to 0 in (b) and (c).

Using deformation potential (DP) theory, the calculated τ is 1.66 × 10^–14^ s for holes and 1.61 × 10^–14^ s for electrons at 300 K for CuBHT-3e (computed parameters are provided in Table S2 in the SI). These values are of the same order of magnitude as, and approximately 2.6 times larger than, the relaxation time obtained for pristine metallic CuBHT (Figure 3d), reflecting the reduced scattering phase space near the band edges in the semiconducting regime. Notably, the τ in CuBHT-3e is significantly closer to the experimentally reported scattering time of $∼41\times 10^{-15}$ s for semiconducting CuBHT^16^ thin films.

The metal-to-semiconductor transition leads to significant changes in charge transport properties. In the metallic regime, the high carrier density results in low *S* and high σ and κ_*e*_, despite increased scattering associated with shorter relaxation times. Upon transition to the semiconducting phase, σ and κ_*e*_ decrease significantly (Figure 4a,b), while *S* shows a strong increase (Figure 3a). While the reduced carrier concentration leads to fewer scattering events and a longer relaxation time (Figure 3d), this increase in τ is not sufficient to compensate for the lower number of charge carriers, resulting in an overall reduction in electrical conductivity. Consequently, the PF of CuBHT-3e is governed by the enhanced Seebeck coefficient up to 350 K, leading to values significantly higher than those of pristine CuBHT (Figure 4c). At higher temperatures, the PF decreases following the trend of σ.

**Figure 4:**
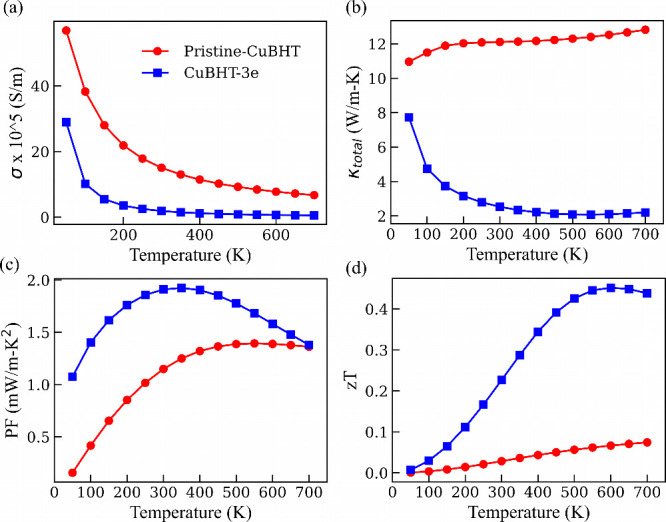
Temperature dependent in-plane thermoelectric parameters, (a) electrical conductivity, (b) total thermal conductivity, (c) thermoelectric power factor (PF) and (d) figure of merit for CuBHT-3e and pristine CuBHT with p-type carrier concentration of 10^20^ cm^–3^.

The *zT* increases rapidly up to 550 K, reaching a maximum value of 0.45 (Figure 4d), before decreasing at higher temperatures. At 300 K, the calculated *zT* reaches 0.23, approximately 1 order of magnitude higher than that of pristine CuBHT, mainly due to the increased *S* and reduced κ_total_. Overall, the obtained PF and *zT* values exceed those reported for other MOFs,^11,13,15^ highlighting redox-driven band engineering as an effective strategy to enhance thermoelectric performance.

It should be noted that the lattice thermal conductivity of the charge-modulated phase was approximated by the value computed for the pristine AA-stacked structure. Because charge injection induces structural buckling and modifies the interlayer bonding network (Figure 2b), the phonon spectrum of the charged phase may differ from that of pristine CuBHT. Moreover, a direct calculation of the dynamical matrix and anharmonic force constants for the charged system was not feasible due to convergence difficulties associated with charged supercells. For the same reason, a complete lattice-dynamical characterization of the charge-modulated phase, including the assessment of its dynamical stability through phonon calculations, remains beyond the scope of the present work and will be addressed in future investigations. Therefore, variations in lattice thermal conductivity, and consequently in the κ_total_ and *zT*, are expected for the semiconducting phase. We observed that the structure of the charged configuration closely resembles the C-stacked structure of CuBHT, which has previously been shown to exhibit significantly lower lattice thermal conductivity than the AA-stacked phase.^21^ Therefore, the obtained zT value may be regarded as a lower bound for estimating the thermoelectric figure of merit.

In order to further tune its band structure and optimize transport properties, we introduce 5% strain along the *a*, *b*, and *ab* directions. The applied strain modifies both the electronic band gap and DOS (Figure S7). In all cases, the band gap changes from direct to indirect and varies between 0.34 and 0.91 eV, with the largest value obtained under 5% tensile strain along the *b* direction (Figure S7). This tunability of the band gap, together with the corresponding changes in band edges, enables further control of the electronic transport coefficients. Thermoelectric properties were evaluated at 300 K under 5% strain in both compressive and tensile conditions. For both carrier types, *S* increases with decreasing carrier concentration, while σ and κ_total_ increase with carrier concentration (Figure 5 for p-type and Figure S8 for n-type carriers). Compared to CuBHT-3e, the *b*-compressive and *ab*-compressive strain configurations exhibit higher S at 10^20^ cm^–3^, while the other strained structures show lower values. This behavior is consistent with the TDF, which decays more rapidly near the Fermi level in these configurations than in the other strained and unstrained CuBHT-3e systems, leading to enhanced *S* (Figure 5). At low carrier concentrations (up to 10^18^ cm^–3^), the onset of charge transport is characterized by a sharp increase in both σ and κ_total_. Above 10^19^ cm^–3^, the system exhibits a more conventional semiconducting behavior. The power factor is mainly governed by σ and increases with carrier concentration for both carrier types. As a result, *zT* follows a similar trend, reaching a maximum value of 0.52 under compressive strain along the *b* and *ab* directions, nearly doubling the value of the unstrained CuBHT-3e structure. The computed *zT* exceeds previously reported values for MOFs at 300 K, highlighting the effectiveness of Fermi level tuning in CuBHT-3e as a strategy for enhancing thermoelectric performance.

**Figure 5:**
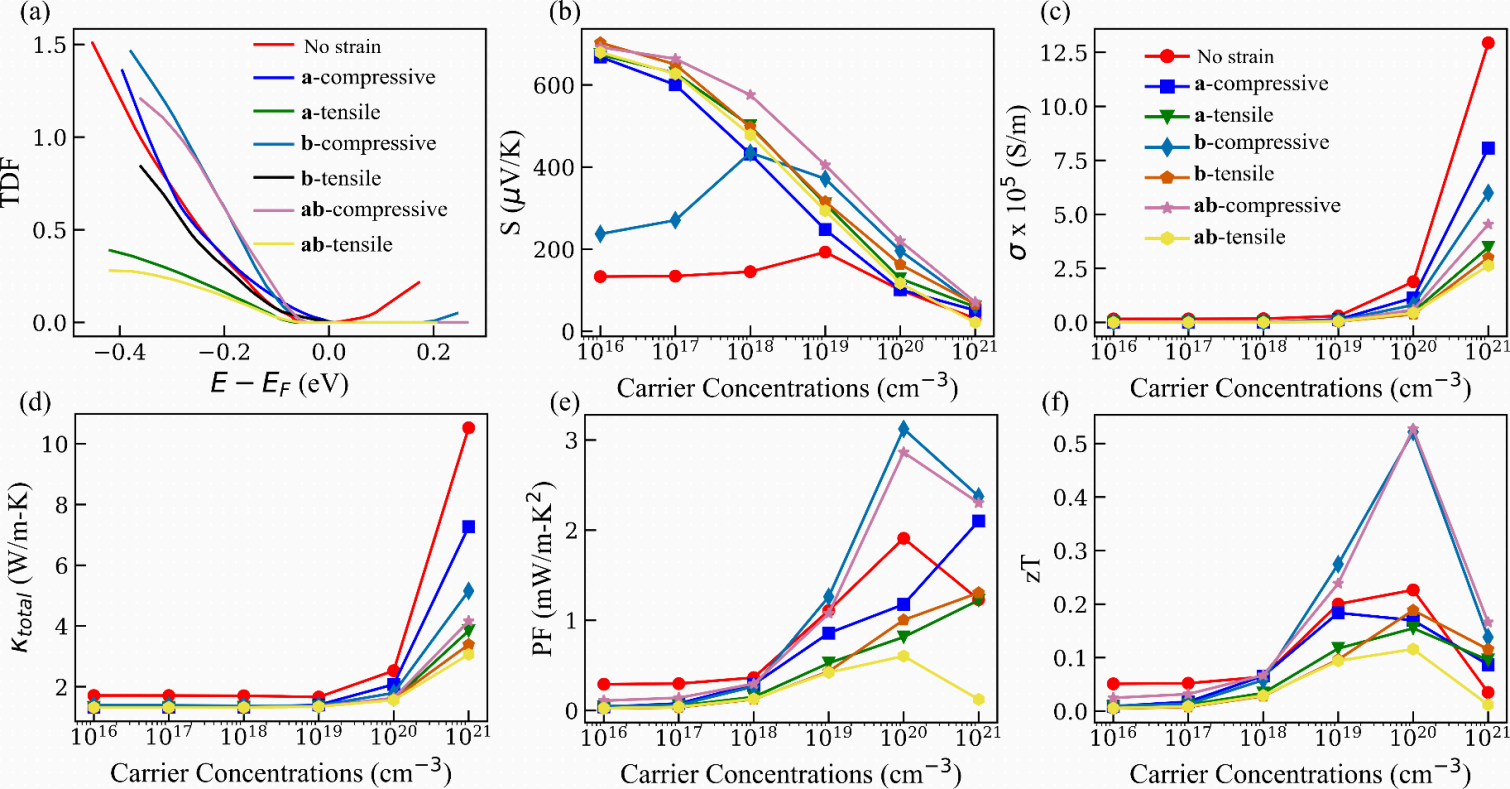
(a) TDF for 5% compressive and tensile strain compared with CuBHT-3e (no strain). (b) *S*, (c) σ, (d) PF, (e) κ_total_ and (f) *zT* for compressive and tensile strain applied along the *a*, *b*, and *ab* directions of the CuBHT-3e. The rapid change in TDF with energy results in high *S* values.

The temperature-dependent thermoelectric properties under compressive strain along the *b* and *ab* directions at a p-type carrier concentration of 10^20^ cm^–3^ are shown in Figure 6. The Seebeck coefficient increases with temperature under biaxial strain, while for uniaxial strain along *b* it decreases above ∼600 K, together with an increase in σ, suggesting a transition toward metallic behavior. Accordingly, *zT* reaches 0.52 at 300 K and increases up to 0.79 at 550 K for both strain configurations. At higher temperatures, *zT* continues to increase under biaxial strain but decreases under uniaxial strain. Considering the thermal stability limit ( ∼750 K),^26^ the maximum reliable *zT* is 0.79 at 550 K. This corresponds to nearly a 2-fold enhancement compared to unstrained CuBHT-3e and an order-of-magnitude improvement over pristine CuBHT, highlighting the effectiveness of the band engineering strategy.

**Figure 6:**
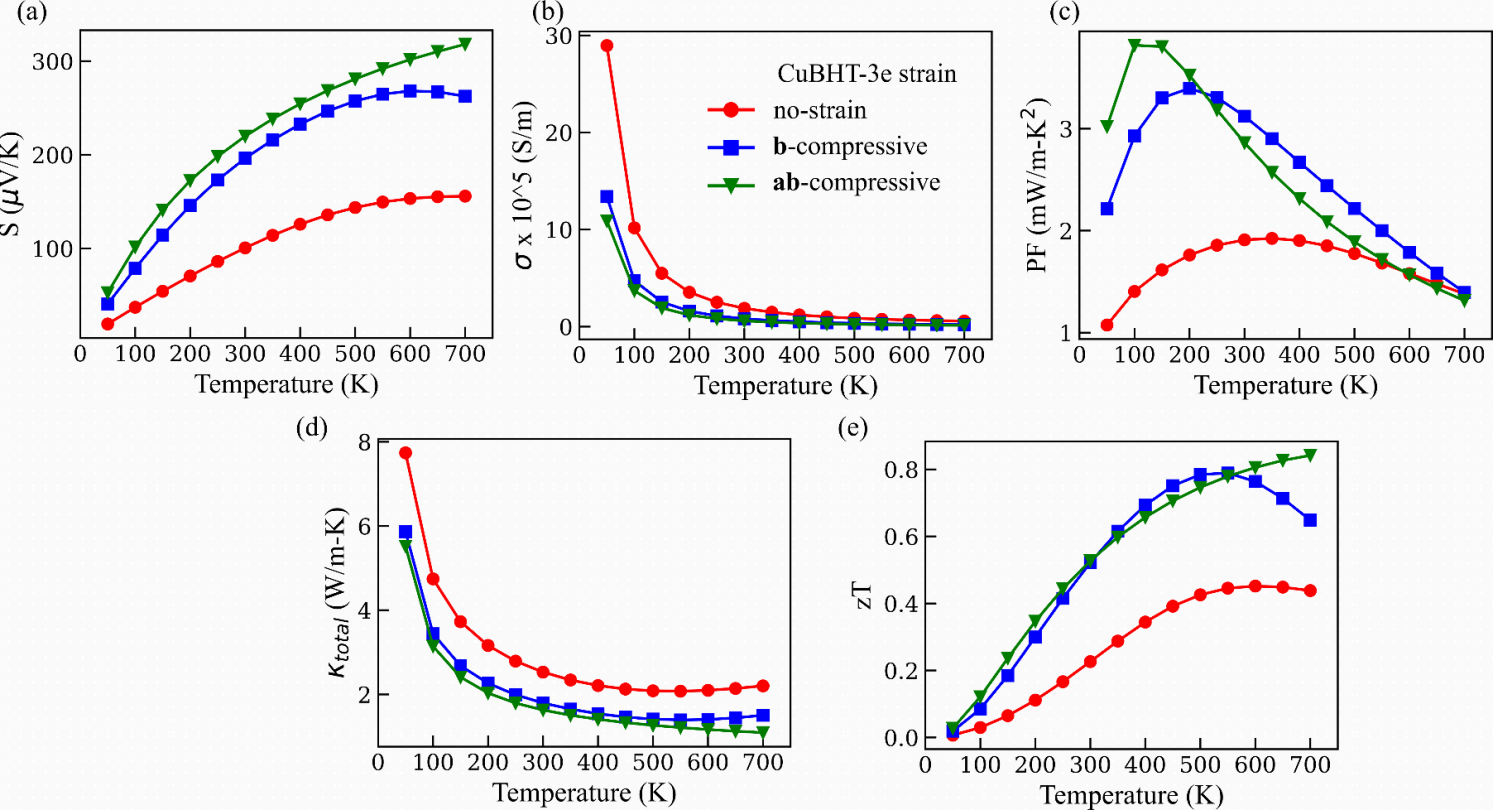
Temperature-dependent thermoelectric properties for p-type carriers with 10^20^ cm^–3^ concentration, (a) *S*, (b) σ, (c) PF, (d) κ_total_ and (e) *zT* for compressive strain applied along the *b* and *ab* directions of the CuBHT-3e.

In summary, this proof-of-concept investigation provides atomistic, first-principles evidence that a band-engineering strategy driven by controlled electrochemical charge modulation represents an efficient design principle to overcome the intrinsic performance limits of conductive two-dimensional metal–organic frameworks in thermoelectric applications. Historically, these systems have suffered from an unfavorable coupling among transport coefficients; their high electrical conductivity, associated with a purely metallic character, drastically suppresses the Seebeck coefficient and induces a dominant electronic contribution to the total thermal conductivity. By systematically evaluating the CuBHT model system, our density functional theory calculations indicate that purely structural modifications (namely, macroscopic elastic strain or lattice vacancy defects) are insufficient to break the underlying metallic landscape, which remains strictly constrained by the pristine orbital symmetry and the high density of states at the Fermi level.

The primary scientific novelty of this investigation lies in unraveling the microscopic coupling between the spatial redistribution of the injected excess charge and the resulting electronic phase transition. The controlled addition of three electrons per unit cell, which mimics the partial reduction of the metal nodes from Cu^2+^ to Cu^+^, does not merely shift the chemical potential deeper into the conduction manifold. More than that, it triggers an extensive electronic and structural rearrangement. The alteration of the charge state mitigates interplanar electrostatic repulsion and drives an out-of-plane structural buckling. This atomistic displacement promotes the hybridization of the copper *d*_*yz*_ orbitals with the sulfur *p*_*z*_ states of adjacent layers, establishing weak interlayer Cu–S covalent interactions along the stacking axis and stabilizing an octahedral-like coordination environment around the copper centers.

From the viewpoint of transport physics, this orbital reconfiguration decouples the bonding and antibonding states that cross the Fermi level in the pristine phase, inducing a metal-to-semiconductor transition accompanied by a band gap opening. The resulting sharp energy dependence of the transport distribution function near the band extrema, driven by the abrupt increase in the density of states, enhances the room-temperature Seebeck coefficient by a factor of 3 relative to the metallic reference. Concurrently, the semiconducting transport regime detrimentally affects the carrier concentration, drastically lowering the electronic thermal conductivity. This allows the system to leverage the ultralow lattice thermal conductivity inherently provided by the soft-bonding character and structural complexity of the MOF architecture.

Finally, the application of a 5% compressive elastic strain enables further tuning of the band dispersion and curvature, inducing a direct-to-indirect band gap transition that modulates the phase space available for electron–phonon scattering processes. This dual electronic and mechanical control elevates the dimensionless figure of merit *zT* to a maximum value of 0.79 at 550 K, approaching the performance of state-of-the-art conventional thermoelectrics. Broadly, these findings establish redox-controlled valency engineering as a powerful methodology for decoupling interdependent transport coefficients in low-dimensional coordinated frameworks, bypassing the limitations of structural disorder and extrinsic defect engineering.

All calculations were performed within the framework of DFT using the Vienna *Ab initio* Simulation Package (VASP) under periodic boundary conditions.^36–38^ The Perdew–Burke–Ernzerhof functional revised for solids (PBEsol) was employed for the exchange–correlation energy, together with DFT-D3 van der Waals corrections.^38,39^ Projector-augmented wave (PAW) pseudopotentials were used with a plane-wave cutoff of 800 eV. Structural relaxations were carried out with force and energy convergence criteria of 10^–2^ eV/Å and 10^–7^ eV, respectively. Brillouin-zone integrations were performed using a reciprocal-space resolution of 0.02 Å^–1^.

Electronic transport properties were computed within the Boltzmann transport framework using the single-mode RTA.^40^ Band velocities and carrier lifetimes were obtained from first-principles calculations and used to evaluate the Seebeck coefficient, electrical conductivity, and electronic thermal conductivity through the transport distribution function,^41^ whose definition is reported in the Supporting Information. Further details are also provided in the SI. For pristine CuBHT, the carrier relaxation time was computed explicitly from electron–phonon scattering rates using Fermi golden rule. The scattering rate for each electronic state was obtained by summing over all phonon modes and final electronic states connected by electron–phonon matrix elements, including both phonon absorption and emission processes. These state-resolved lifetimes were then averaged over the relevant states around the Fermi level and used in the evaluation of the transport coefficients. This treatment retains the momentum- and band-resolved character of the electron–phonon scattering and is therefore more appropriate for metallic pristine CuBHT than a constant relaxation time approximation.^41^ For charge-modulated systems, transport occurs in a semiconducting regime near the band extrema, where scattering is dominated by acoustic phonons. In this case, DP theory^42^ was employed within the constant relaxation time approximation (CRTA) to estimate the carrier relaxation time. The relaxation time was obtained from τ = μm*/*e*, where the carrier mobility μ was derived from the deformation potential and elastic constants, as detailed in the Supporting Information. Finally, electronic transport coefficients were evaluated using a dense 16 × 16 × 32 ***k***-point grid. The lattice thermal conductivity used for the calculation of *zT* was taken from our previous work.^21^ Additional computational details are provided in the Supporting Information.

## Supplementary Material



## Notes

Additional methodology for the computation of thermoelectric parameters, the electronic band structure and temperature-dependent thermoelectric parameters for defect- and strain-induced structures, hybrid functional band structure for the charged structure CuBHT-3e, along with thermoelectric properties for both CuBHT-3e and strained CuBHT-3e, and a table summarizing the parameters used for the computation of relaxation time based on deformation potential theory, as well as the charges on pristine and charged CuBHT (PDF)
