# Supplementary material for: Band Engineering via Charge Modulation Drives High Thermoelectric Performance in Conductive MOFs
Source: J Phys Chem Lett. 2026 Jul 17;17(30):8624–33. doi: 10.1021/acs.jpclett.6c01808 (PMC13430686; doi:10.1021/acs.jpclett.6c01808)
Supplement: Supplementary file 1 [file jz6c01808_si_001.pdf]

Band engineering via charge modulation drives high thermoelectric performance in conductive MOFs

Hardik L. Kagdada[1], R. Dettori[1], L. Colombo[1], C. Melis[1]
[1]Department of Physics, University of Cagliari, Monserrato, CA, 09042, Italy
*Corresponding Author: claudio.melis@dsf.unica.it; hlkagdada@gmail.com

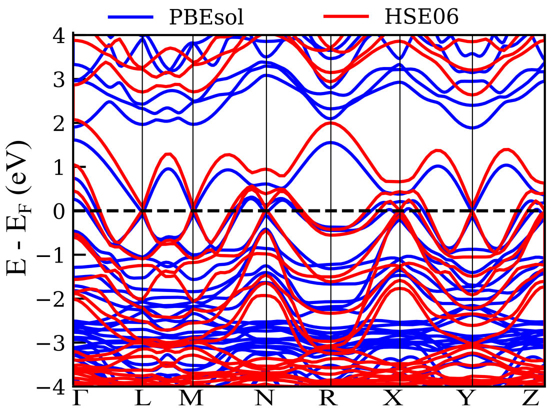

Figure S1: A comparison of the electronic band structures calculated using PBEsol+HSE06 and PBEsol functionals for pristine CuBHT demonstrates that the metallic character is retained when the HSE06 hybrid functional is applied.

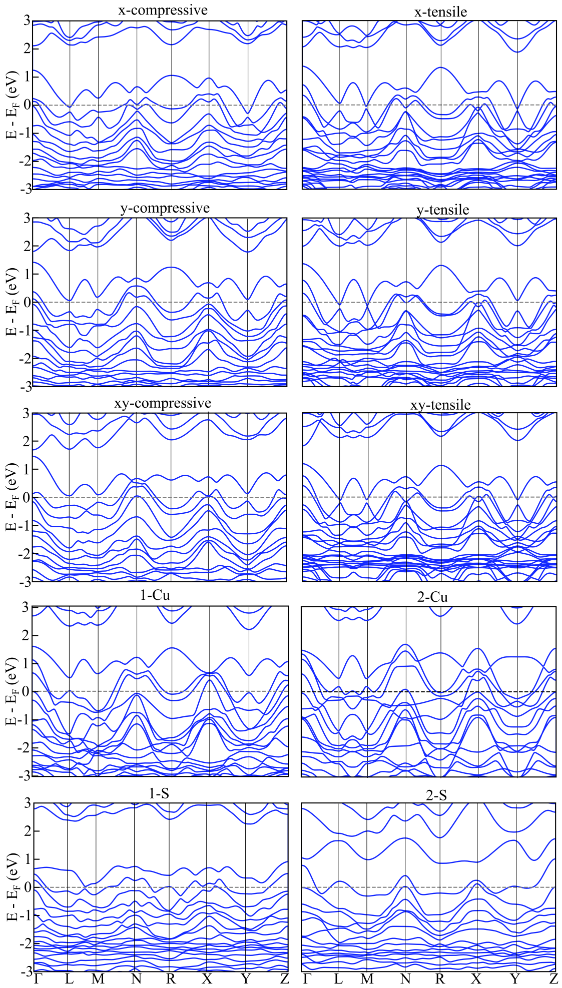

Figure S2: Computed electronic band structure for strain and defect structures. All structures show metallic character.

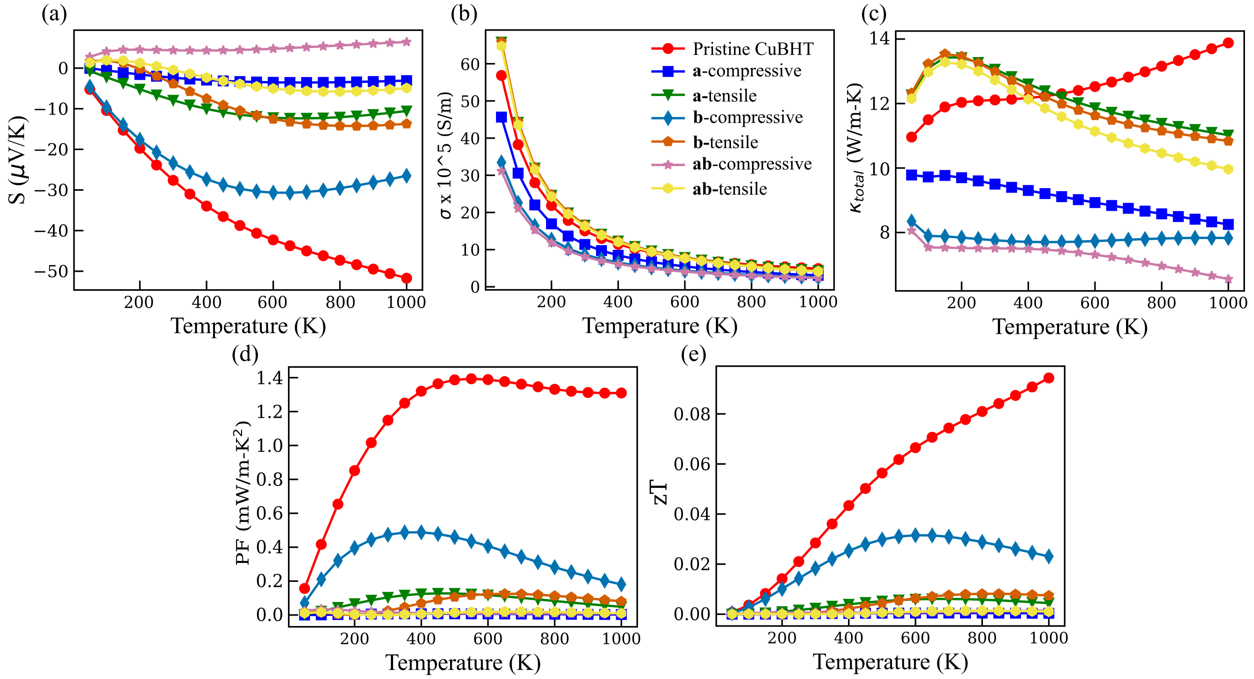

Figure S3: Thermoelectric parameters for 5% compressive and tensile strain along the a, b, and ab directions of the CuBHT structure: (a) Seebeck coefficient (S), (b) electrical conductivity, (c) total thermal conductivity (κ_{total}), (d) power factor, and (e) figure of merit zT as a function of temperature for p-type carriers with carrier concentration of 10^{20} cm^{-3}.

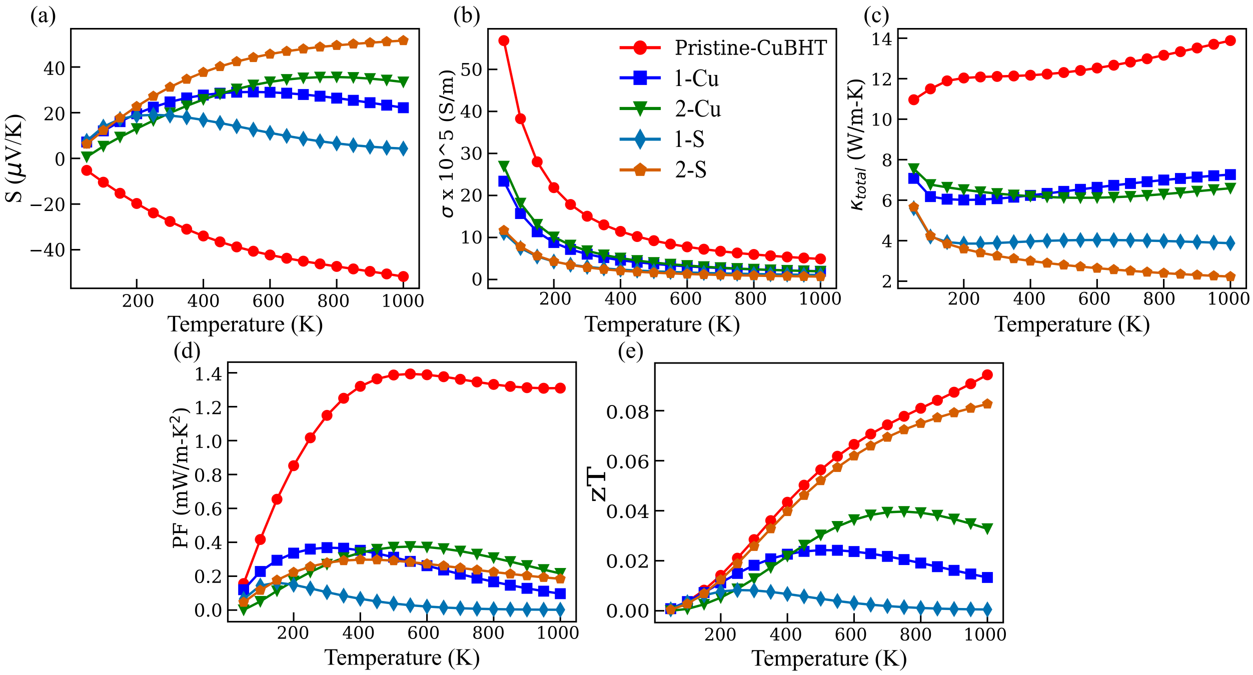

Figure S4: Same as Figure S2, for Cu and S defects in CuBHT.

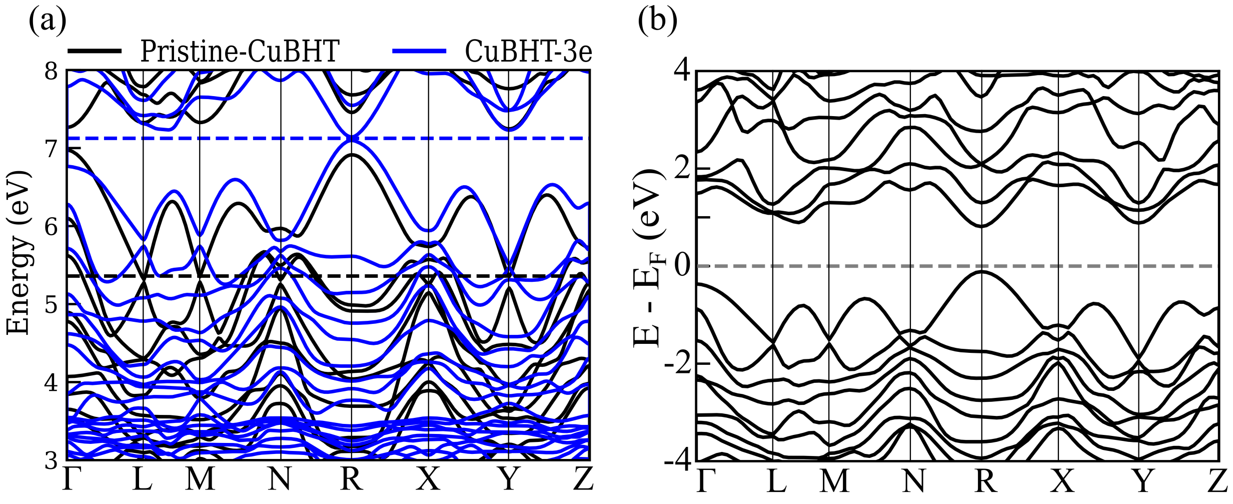

Figure S5: (a) Comparison of the electronic band structure of pristine CuBHT and CuBHT-3e. Black and blue dashed lines indicate the Fermi level for pristine CuBHT and CuBHT-3e, respectively. Band structures are computed at the PBEsol level. (b) Electronic band structure computed with the DFT+HSE06 functional for CuBHT-3e, showing a direct band gap of 0.9 eV at the high-symmetry point R. The Fermi level is set to 0 eV.

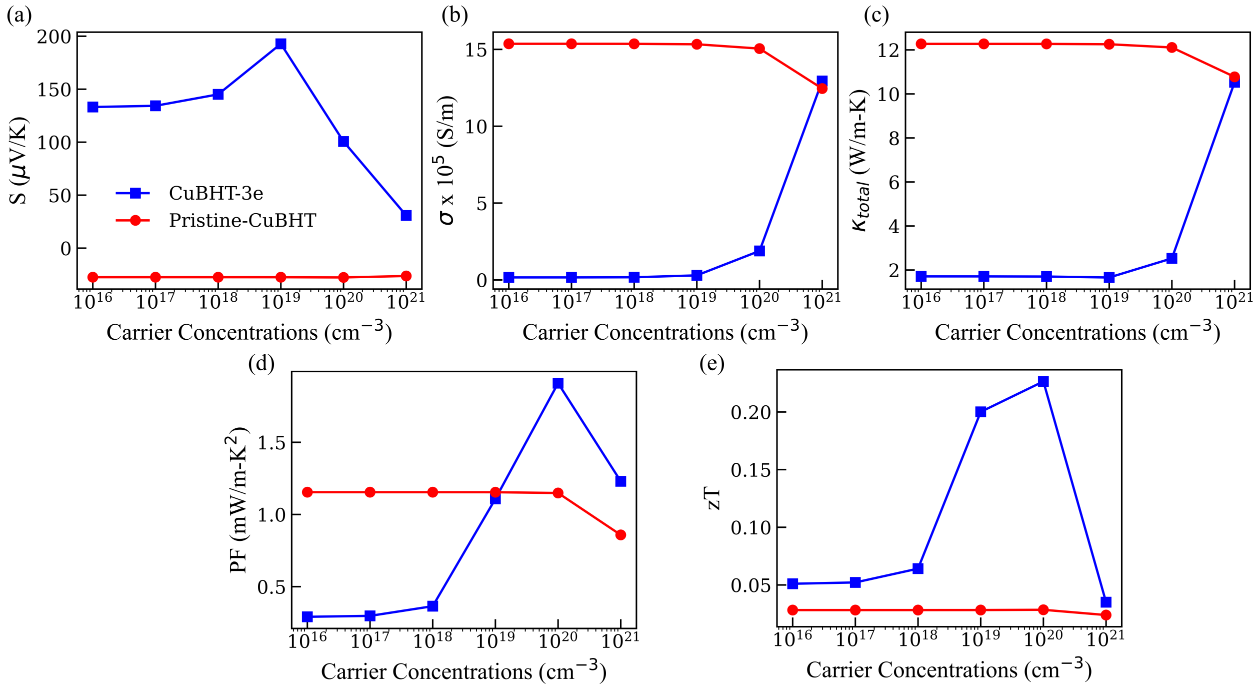

Figure S6: Comparison of thermoelectric parameters at 300 K between pristine CuBHT and CuBHT-3e: (a) Seebeck coefficient (S), (b) electrical conductivity, (c) total thermal conductivity (κ_{total}), (d) power factor, and (e) figure of merit zT as a function of p-type carrier concentration.

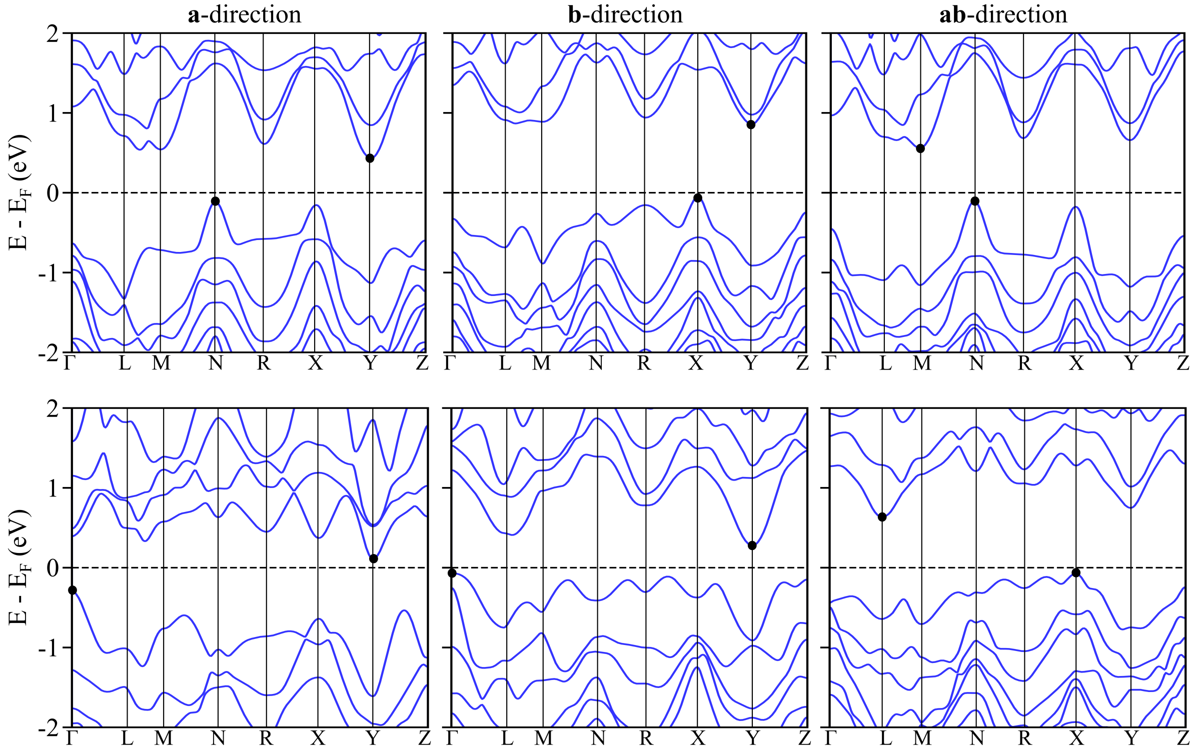

Figure S7: Computed electronic band structure for strained CuBHT-3e with 5% compresive and tensile strain applied along the along the a, b, and ab directions

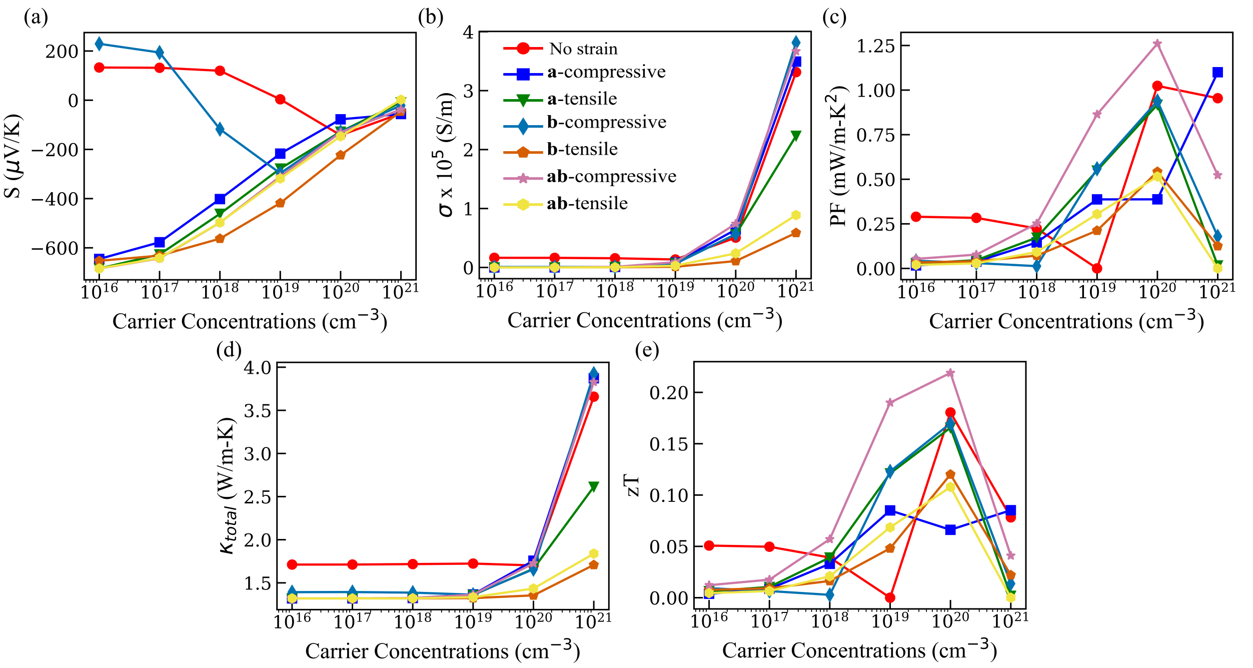

Figure S8: Strain-induced thermoelectric parameters at 300 K for n-type carriers. The 5% strain is applied along the a, b, and ab directions of the CuBHT-3e structure: (a) Seebeck coefficient (S), (b) electrical conductivity, (c) power factor, (d) total thermal conductivity (κ_{total}), and (e) figure of merit zT as a function of carrier concentration.

Tables

Table S1: Bader charges (in units of e) for pristine CuBHT and CuBHT-3e. Negative differences indicate an increase in negative charge in CuBHT-3e.

Atom	Pristine	CuBHT-3e	Difference
S	0.019	-0.145	-0.164
S	0.019	-0.272	-0.291
S	0.019	-0.145	-0.164
S	0.019	-0.272	-0.291
S	-0.068	-0.262	-0.194
S	-0.068	-0.262	-0.194
C	-0.093	-0.380	-0.287
C	-0.062	-0.008	0.054
C	-0.098	-0.380	-0.282
C	-0.063	-0.012	0.051
C	-0.139	-0.369	-0.230
C	-0.140	-0.369	-0.229
Cu	0.280	-0.062	-0.342
Cu	0.209	-0.039	-0.248
Cu	0.166	-0.025	-0.191

Table S2: Effective mass (m^*), deformation potential (E_1), and elastic constant (C) used to compute the carrier relaxation time.

Carrier	m^* (m_0)	E_1 (eV)	C (GPa)
Electrons	1.91	9.11	101.66
Holes	1.42	11.17	101.66

Extended Computational Details

Electronic transport properties were computed within the semiclassical Boltzmann transport equation in the relaxation-time approximation [1]. The Seebeck coefficient S, electrical conductivity σ, and electronic thermal conductivity κ_e are expressed as

$$S = \frac{1}{eT} \frac{\int d\varepsilon\, \mathcal{T}(\varepsilon)(\varepsilon - \mu)\left(-\frac{\partial f}{\partial \varepsilon}\right)}{\int d\varepsilon\, \mathcal{T}(\varepsilon)\left(-\frac{\partial f}{\partial \varepsilon}\right)}, \tag{1}$$

$$\sigma = \int d\varepsilon\, \mathcal{T}(\varepsilon)\left(-\frac{\partial f}{\partial \varepsilon}\right), \tag{2}$$

$$\kappa_e = \frac{1}{T} \int d\varepsilon\, \mathcal{T}(\varepsilon)(\varepsilon - \mu)^2 \left(-\frac{\partial f}{\partial \varepsilon}\right). \tag{3}$$

Here μ is the chemical potential, and $f(\varepsilon)$ is the Fermi–Dirac distribution function. The transport distribution function $\mathcal{T}(\varepsilon)$ describes the energy-resolved contribution of electronic states to transport [1] and is defined as

$$\mathcal{T}(\varepsilon) = \frac{e^2}{N_k \Omega} \sum_{n\mathbf{k}} \tau_{n\mathbf{k}} \, \mathbf{v}_{n\mathbf{k}} \otimes \mathbf{v}_{n\mathbf{k}} \, \delta(\varepsilon_{n\mathbf{k}} - \varepsilon), \tag{4}$$

where N_k is the number of k-points, Ω is the unit cell volume, $\tau_{n\mathbf{k}}$ is the carrier relaxation time, $\mathbf{v}_{n\mathbf{k}}$ is the band velocity, and $\varepsilon_{n\mathbf{k}}$ is the band energy.

For pristine CuBHT, the carrier relaxation time was computed from electron–phonon interactions using Fermi's golden rule [2],

$$\frac{1}{\tau_{n\mathbf{k}}} = \frac{2\pi}{\hbar} \sum_{m\nu\mathbf{q}} w_{\mathbf{q}} \left| g^{\nu\mathbf{q}}_{n\mathbf{k},m\mathbf{k}+\mathbf{q}} \right|^2 \Big[(n_{\nu\mathbf{q}} + 1 - f_{m\mathbf{k}+\mathbf{q}}) \delta(\varepsilon_{n\mathbf{k}} - \varepsilon_{m\mathbf{k}+\mathbf{q}} - \hbar\omega_{\nu\mathbf{q}}) + (n_{\nu\mathbf{q}} + f_{m\mathbf{k}+\mathbf{q}}) \delta(\varepsilon_{n\mathbf{k}} - \varepsilon_{m\mathbf{k}+\mathbf{q}} + \hbar\omega_{\nu\mathbf{q}}) \Big], \tag{5}$$

where $w_{\mathbf{q}}$ is the Brillouin-zone weight, $n_{\nu\mathbf{q}}$ is the phonon occupation, and $\omega_{\nu\mathbf{q}}$ is the phonon frequency.

The electron–phonon matrix elements are given by The electron–phonon matrix elements are given by

$$g^{\nu\mathbf{q}}_{n\mathbf{k},n'\mathbf{k}'} = \langle \psi_{n\mathbf{k}} | \partial_{\nu\mathbf{q}} V | \psi_{n'\mathbf{k}'} \rangle, \tag{6}$$

where $\psi_{n\mathbf{k}}$ are the Kohn–Sham wavefunctions and $\partial_{\nu\mathbf{q}} V(\mathbf{r})$ is the variation of the self-consistent potential associated with the phonon mode $(\nu, \mathbf{q})$.

For charge-modulated systems, transport occurs in a semiconducting regime near the band extrema, where scattering is dominated by acoustic phonons. In this case, deformation-potential (DP) theory [3] was employed within the constant relaxation time approximation (CRTA).

The relaxation time was obtained from $\tau = \mu m^*/e$, with the carrier mobility given by

$$\mu = \frac{(8\pi)^{1/2} e \hbar^4 C^{3D}}{3 (m^*)^{3/2} (k_B T)^{5/2} E_1^2}, \tag{7}$$

where E_1 is the deformation potential constant and C^{3D} is the elastic constant along the transport direction. The deformation potential was extracted from band-edge shifts under $\pm 1\%$ strain, while C^{3D} was obtained from the second derivative of the total energy with respect to strain.

References

[1] Georg K. H. Madsen and David J. Singh. Boltztrap. a code for calculating band-structure dependent quantities. *Computational Physics Communications*, 175(1):67–71, 2006.

[2] F. Giustino. Electron-phonon interactions from first principles. *Reviews of Modern Physics*, 89:015003, 2017.

[3] J. Bardeen and W. Shockley. Deformation potentials and mobilities in non-polar crystals. *Physical Review*, 80:72–80, 1950.
